# A Stress Management App Intervention for Cancer Survivors: Design, Development, and Usability Testing

**DOI:** 10.2196/formative.9954

**Published:** 2018-09-06

**Authors:** Elin Børøsund, Jelena Mirkovic, Matthew M Clark, Shawna L Ehlers, Michael A Andrykowski, Anne Bergland, Marianne Westeng, Lise Solberg Nes

**Affiliations:** 1 Center for Shared Decision Making and Collaborative Care Research Division of Medicine Oslo University Hospital Oslo Norway; 2 Department of Psychiatry and Psychology College of Medicine and Science Mayo Clinic Rochester, MN United States; 3 Department of Behavioral Science College of Medicine University of Kentucky Lexington, KY United States; 4 Institute of Clinical Medicine Faculty of Medicine University of Oslo Oslo Norway

**Keywords:** stress management, mindfulness, cancer, eHealth, mHealth, mobile apps, development, usability, user-centered design, mobile phones

## Abstract

**Background:**

Distress is prevalent in cancer survivors. Stress management interventions can reduce distress and improve quality of life for cancer patients, but many people with cancer are unfortunately not offered or able to attend such in-person stress management interventions.

**Objective:**

The objective of this study was to develop an evidence-based stress management intervention for patients living with cancer that can be delivered electronically with wide reach and dissemination. This paper describes the design and development process of a technology-based stress management intervention for cancer survivors, including the exploration phase, intervention content development, iterative software development (including design, development, and formative evaluation of low- and high-level prototypes), and security and privacy considerations.

**Methods:**

Design and development processes were iterative and performed in close collaboration with key stakeholders (N=48). In the exploration phase, identifying needs and requirements for the intervention, 28 participants gave input, including male and female cancer survivors (n=11) representing a wide age range (31-81 years) and cancer diagnoses, healthcare providers (n=8) including psychosocial oncology experts, and eHealth experts (n=9) including information technology design and developers. To ensure user involvement in each phase various user-centered design and service design methods were included, such as interviews, usability testing, and think aloud processes. Overall, participants were involved usability testing in the software development and formative evaluation phase, including cancer survivors (n=6), healthy volunteers (n=7), health care providers (n=2), and eHealth experts (n=5). Intervention content was developed by stress management experts based on well-known cognitive behavioral stress management strategies and adjusted to electronic format through multiple iterations with stakeholders. Privacy and security issues were considered throughout.

**Results:**

The design and development process identified a variety of stakeholder requirements. Cancer survivors preferred stress management through a mobile app rather than through a personal computer (PC) and identified usefulness, easy access, user friendliness, use of easily understandable language, and many brief sections rather than longer ones as important components of the intervention. These requirements were also supported by recommendations from health care providers and eHealth experts. The final intervention was named *StressProffen* and the hospital Privacy and Security Protection Committee was part of the final intervention approval to also ensure anchoring in the hospital organization.

**Conclusions:**

Interventions, even evidence-based, have little impact if not actively used. This study illustrates how user-centered design and service design can be applied to identify and incorporate essential stakeholder aspects in the entire design and development process. In combination with evidence-based concepts, this process facilitated development of a stress management intervention truly designed for the end users, in this case, cancer survivors.

**Trial Registration:**

ClinicalTrials.gov NCT02939612; https://clinicaltrials.gov/ct2/show/NCT02939612 (Archived at WebCite at http://www.webcitation.org/71l9HcfcB)

## Introduction

Cancer diagnoses and subsequent treatments can be disruptive and traumatic, often accompanied by a multitude of stressors for the cancer patients and their support network [1-3]. Uncertainty of outcome and medical procedures with adverse side effects are not uncommon, and although people differ widely in how they experience and cope with such challenges, cancer-related distress, including worry, anxiety, depression, and reduced quality of life (QoL), are prevalent [2,4,5]. Fortunately, cancer survival rates are improving, but survivorship is accompanied by long-term health challenges, and many survivors struggle to cope and maintain a positive QoL [6,7].

Psychosocial cognitive behavioral stress management interventions are usually delivered face-to-face, either as individual or group interventions. They are widely recognized as effective, well documented, structured, and multidisciplinary, focusing on specific strategies to improve physical, social, emotional, functional, and overall well-being [4,8-13]. The interventions are based on the cognitive behavioral therapeutic models and address factors related to cognitive, emotional, and behavioral aspects that might enhance coping, including but not limited to educational information, problem-solving skills, self-care strategies, thought awareness and mood management, health behavior change, communication strategies, social support, and relaxation and mindfulness training. Such psychosocial cognitive behavioral stress management interventions have been shown to facilitate psychological adaptation to cancer, including reducing distress, anxiety, negative affects, and depression, as well as improving QoL in cancer patients and survivors [4,8-10,12,13]. These positive findings are also supported by reviews and meta-analyses [14-20]. Some of the psychosocial cognitive behavioral stress management interventions for cancer patients and survivors have even been shown to have beneficial effects on immune markers [8,21,22], and there are indications that cancer recurrence and survival rates may also be positively affected [20,23].

Unfortunately, face-to-face psychosocial interventions are not always offered or easily available to the cancer survivor. In addition, patients with cancer face many demands and stressors, often feel overwhelmed, and may be reluctant to take on the additional commitment of attending and engaging in psychosocial intervention programs [13]. If psychosocial interventions are unavailable or attending in-person services appears too challenging despite unmet needs, innovative thinking is needed about how psychosocial challenges can be addressed and coping skills supported in a format that is appealing and available to cancer survivors.

With the rapid advance of technology, the evolving concept of eHealth encompasses a range of systems or services in a novel cross-section between medicine, health care, and information technology. eHealth solutions have the potential to provide support anytime and anywhere, which again can facilitate ways to reach, service, and intervene when most needed or convenient for the cancer trajectory [24]. Development and testing of psychosocial eHealth interventions programs for cancer survivors are still at an early stage, and evidence of the effects of eHealth interventions so far are mixed. A meta-review identified eHealth interventions to have positive links to perceived support, knowledge, and information competence among cancer patients but found inconsistent or lacking results for areas such as psychological well-being and QoL [25]. Another systematic review examining the use of Web-based resources for adult cancer survivors also found efficacy to vary with some positive effects on QoL and related psychosocial factors but overall mixed efficacy and limited duration of benefit [26]. Examples of promising findings include improved self-efficacy for coping with cancer through the use of a Web-based stress management workbook for breast cancer patients [27], improved QoL and physical activity for breast cancer survivors through use of a Web-based portal [28], and improved QoL and reduced distress for newly diagnosed patients with cancer through use of a Web-based structured Web-based stress management program guided by psychologists [29]. There are indications that the therapist-guided eHealth interventions may be more effective than self-guided interventions [30]. With promising yet mixed results, some investigators and clinicians have called for more use of evidence-based interventions and rigorous monitoring of program impact for future eHealth intervention research in cancer [26]. Even though several studies report on results from psychosocial interventions delivered via the internet, Web, or Web-based sources, few, if any, have explored building and testing app-based psychosocial interventions for cancer survivors. A recent review of available breast cancer apps concluded that most such apps appear to be lacking evidence and an evidence base and that health care providers, not just start-up companies and entrepreneurs, should be included in such developments [31].

This study reports on the design and development of a technology- and app-based stress management intervention for cancer survivors. The study combined well-established cognitive behavioral stress management concepts shown to be effective for patients with cancer in face-to-face interventions [4,8-10,12,13,32,33] with a user-centered design approach to ensure that the intervention was designed in line with users’ needs and context of use. The main philosophy behind the user-centered design approach is to include users in the design and development process and allow system end users to influence how the product takes shape [34,35]. To support this process, the user-centered design provides a variety of methods enabling user involvement in different phases of development with different levels of user engagement. Service design is another approach to system design, focusing on service development. This approach focuses on the entire ecosystem and experiences around it (eg, how it is used, by whom, when, and where) rather than the end product alone [36]. This study combined the user-centered and service design approaches to enable stakeholder involvement throughout the entire design and development process. This was done to ensure intervention alignment with the needs and requirements of cancer survivors and health care professionals alike. The ultimate goal was to have an end product that is both user friendly and useful and also engaging and motivating and that fits into the bigger context of the everyday life and challenges of people living with cancer. Cancer survivors, health care providers, including psychosocial oncology specialists, and eHealth experts were actively involved in the entire process.

## Methods

### Overview

The design and development process encompassed a multidisciplinary approach and continuous systematic evaluation throughout, as recommended in the Center for eHealth Research and Disease Management comprehensive roadmap approach to improve the uptake and impact of eHealth technologies [37]. The intervention development work was led by the study principal investigator, who is a clinical psychologist with health psychology specialization and longstanding experience in psychosocial oncology, stress management, and cognitive behavioral treatment approaches for medical patients. The multidisciplinary project team had weekly meetings during the design and development phase and consisted of experts in stress management, psychosocial oncology, eHealth research, and information technology (IT) developers as well as a designer and content specialists. User-centered and service design methodologies were applied to ensure user involvement throughout the entire design and development process. Patient representatives, health care providers, including psychologists and cancer nurses, and security experts were consulted throughout.

**Figure 1:**
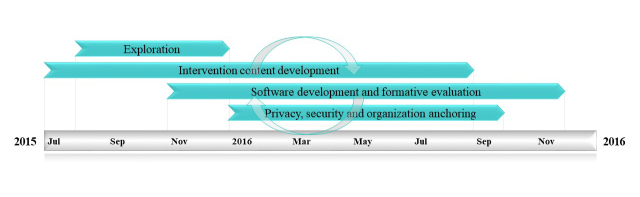
Development timeline.

The stress management intervention program was developed in iterative processes, as shown in Figure 1, through a combination of exploration phase: input from user representatives (ie, cancer survivors), health care providers, and eHealth experts including designers and developers; intervention content development: identified and adjusted from the evidence-based cognitive behavioral stress management concept; iterative software development and formative evaluation; and (4) privacy, security, and organization anchoring considerations.

### Exploration

#### Input from User Representatives (Cancer Survivors)

To identify user needs and requirements of the technology-based stress management intervention, people with any type of cancer diagnosis were invited to participate in individual interviews. They were recruited through the Oslo University Hospital, Oslo, Norway, and collaborating networks, social media such as Facebook, and through the Norwegian Cancer Society. Inclusion criteria for participation in interviews were as follows: diagnosed with cancer or cancer survivors, 18 years or older, and fluent in the Norwegian language. Potential participants were given oral and written information about the study and if interested in participation, they were provided written informed consent prior to study enrollment.

Participants in this phase could choose if they wanted to be interviewed face-to-face or by telephone. They were asked about challenges in their health situation, their use of technology (eg, smart phones; tablets; or personal computers [PCs]) and health technology (eg, websites and apps), their requirements for the use of eHealth interventions, and any suggestions they might have for the design and development of an electronic stress management intervention. Interviews were conducted by 2 representatives from the research team and audiotaped, then transcribed focusing on essential parts, and analyzed using content analysis [38].

#### Input from Health Care Providers and eHealth Experts

Supplementing patient user input, health care providers (ie, registered nurses and clinical psychologists) with longstanding experience working with cancer patients were invited to act as consultants and give input on intervention development, including design, intervention content, and ideas on how and when a stress management intervention could be offered to cancer survivors. eHealth experts collaborating with the project team with extensive experience in development of self-management apps for chronically ill people were also invited to give input on how the intervention could best be designed and delivered to optimize presentation, engagement, adherence, and potential effect.

### Intervention Content Development

#### Evidence-Based Content Development

A major goal of this study was to identify evidence-based factors and areas from well-known cognitive behavioral stress management strategies and then synthesize and adapt these into a new technology-based stress management intervention for cancer survivors. When identifying concepts and factors to develop content for this intervention, potential underlying mechanisms, including likely mediators such as psychosocial resources, were considered to best integrate theory, research, and practice in support of cancer survivors [8,10,15,39-41]. Such integrations have the potential to address a wide array of issues and challenges faced by many patients with cancer.

#### Adjustment to Electronic Format

Intervention content was adjusted to an electronic format to facilitate intuitive use for the cancer survivors. Adjustments were made in 6 iterations to ensure easy language, short sentences, and focus on clear content for small screens.

### Software Development and Formative Evaluation

#### Iterative Development and Low-Fidelity Prototypes

**Figure 2:**
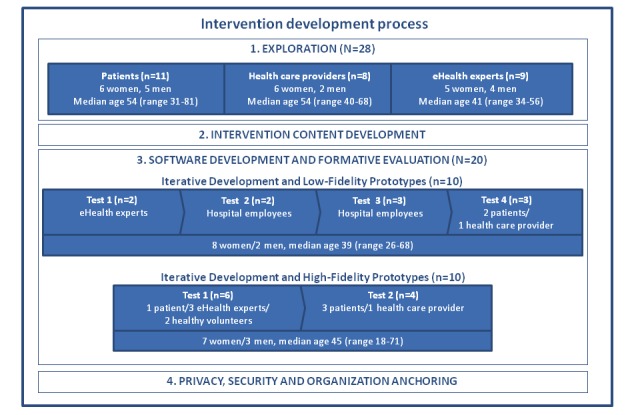
Intervention development process and participants.

Based on needed content adjustments and stakeholder input identified in the exploration phase, the first low-fidelity prototype version of the software was developed. This initial paper prototype consisted of the start page, the menu page, and screens presenting the first intervention module design and content. Next, the prototype was evaluated via 4 consecutive iterations and refined and adjusted based on user feedback, as seen in Figure 2.

In the first iteration, eHealth experts tested and gave feedback on the prototype to ensure that the intervention program was logically built and would meet the stakeholder requirements. After minor adjustments, the paper prototype was implemented into an electronic tool for testing of paper prototypes by simulating the app idea (Prototyping on Paper app by Marvel) [42]. Hospital-employed healthy volunteers then tested the prototype in the second iteration and provided feedback. A third iteration, including hospital-employed healthy volunteers, resulted in minor adjustments, and the prototype was deemed ready for usability testing with cancer survivors. In the final iteration, 2 female cancer survivors and one health care provider (psychologist) tested the final version of the low-fidelity prototype. Healthy volunteers and cancer survivors were given oral and written information about the study and provided written informed consent prior to user testing.

During testing, the participants were asked by a facilitator to navigate through the prototype and describe their actions. All movements from elbow to fingertips were filmed; using the think aloud method, a research assistant asked follow-up questions as the module testing progressed [43]. An observer made notes and summarized the input from notes and the video into a report that subsequently provided recommendations for prototype adjustments. Following the development of a final low-fidelity prototype, the high-fidelity prototype development started.

#### Iterative Development and High-Fidelity Prototypes

Usability refers to the ability of an app to be understood, learned, used, and also be attractive to intended users under specific conditions of use [44]. During the high-fidelity prototype development, the actual software start page, menu page, and first intervention module were built. To ensure the usability and user need fit of the high-fidelity prototype, a new round of iterative testing and evaluation was performed. To incorporate the gender and age perspective, testing encompassed male and female participants aged 18-70 years old. Six participants were involved in this iteration, as elaborated in Figure 2.

Following adjustments, the new high-fidelity prototype version was then tested by 3 cancer survivors and a health care provider (a psychologist). During this high-fidelity prototype testing, participants were asked to go through the entire first intervention module and comment on their movements. Testing was again performed through the filming of movements (elbow to fingertip), follow-up questions (eg, Can you tell me why you are doing this? Can you find the exercise overview/my page/settings?), note taking, and a summarizing report. Resulting stakeholder feedback data were again used to evaluate, refine, iteratively adjust, and upgrade the prototype. All functionalities, content descriptions, and modules were tested, and user feedback was obtained. Because this is a self-help program with extensive and, at times, repetitive cognitive behavioral content, all functionalities, but not all content paragraphs, were user tested.

### Privacy, Security, and Organization Anchoring

One major stakeholder in this project is the hospital (ie, organization) where the intervention is developed. To plan for postproject implementation, the initial project idea and plan were registered at the hospital innovation unit. This unit provides advice for potential commercialization and anchoring in the organization and welcomes all innovative ideas. It is a requirement to register all innovations at the hospital innovation unit. To ensure that all privacy and security requirements were considered and attended to for the project, the hospital Privacy and Security Protection Committee was consulted at a very early stage. Topics discussed were options to store personal and health-related data in the solution, local versus server data storage, user authentication requirements, and other related issues. All procedures, including the informed consent process, were conducted in accordance with existing ethical standards [45]. The study was approved by the hospital Privacy and Security Protection Committee. It describes development of the intervention that will be tested in a Randomized Controlled Trial as registered at ClinicalTrials.gov (NCT02939612).

## Results

### Exploration

#### Participants

Cancer survivors (n=11) with a variety of cancer diagnoses participated in individual interviews giving input on daily challenges and support during cancer treatment, their use of technology and health information, and needs and requirements for a stress management intervention program. Participants were women (6/11, 55%) and men (5/11, 45%) aged 31-81 years old (median 54 years). Time since cancer diagnosis was 0-14 years (median 4.7 years). Most participants (8/11, 73) chose telephone interviews. The participants represented a variety of demographic factors, including gender, age, and diagnosis. Few new topics emerged after the first 2/3 of interviews, and recruitment was therefore completed at n=11 (saturation).

Health care providers (n=8), 3 cancer nurses and 5 psychologists working within psychosocial oncology, eHealth experts (n=9), 3 research scientists, 2 content experts, a designer, and 3 developers also gave input on when a stress management intervention could or should be offered to cancer survivors and how the intervention content could best be presented and delivered.

#### Input from User Representatives (Cancer Survivors)

Participants reported a broad spectrum of everyday challenges during cancer treatment including stress, loss of memory, confusion about the situation, sleep disturbance, depression, worries, new self-image, fatigue, pain, stiffness, and being isolated from work and society or social settings. Their main sources of social support during treatment were reported to be family and friends, but they also reported support from health care providers, including nurses, psychologists, and general practitioners, as well as peer support through the internet.

All participants had access to a smartphone and a PC and rated their user experience as medium to high. The majority (7/11, 64%) had access to a tablet. They all used apps, installed either by themselves or by their children, and they used the internet at least once a day. Mostly, they reported using the smartphone for practical issues, communication, or distraction. Those who used health apps preferred relaxation programs. Others had installed health apps but had limited engagement, stating that they forgot to use them or lost interest after a while.

User-reported needs and requirements for the use of an eHealth intervention can be summarized in 3 key areas. The app had to fulfill their needs as cancer survivors, be easily accessible, and be intuitive and easy to use. When asked about the potential use of a PC for stress management, a majority of participants reported associating use of PC with work. When relaxing, they preferred to use either their tablet or their smartphone. Some of the oldest participants (n=4; age range 51-81 years) anticipated that they would not use a stress management program on a smartphone owing to difficulties with a small screen. Two participants preferred to use their smartphone, however, because it was “always around” 4 participants preferred to use an app compared with a static website because they expected an app to be more easily accessible. Preferred presentation of the intervention content was a combination of sound files, text, and a video. Some participants expected that they would like to have a possibility to read more about the topics.

#### Input from Health Care Providers and eHealth Experts

It was advised that patients should wait to utilize the intervention until minimum 1 month after receiving a cancer diagnosis and at least a few weeks after the initial cancer treatment had started. This is often a very challenging time with patients mainly focusing on processing the new situation and getting started with cancer treatment as soon as possible. The intervention could be offered at out-patient clinics, radiation treatment clinics, and the learning and mastery units or psycho-oncology units. Male patients were described as those who seldom attend group interventions for stress management, and the design team was advised to focus on a design that could appeal to male as well as female users.

Because cancer diagnosis and subsequent treatment are often accompanied by lack of energy, problems with concentration, stress, and distress, it was suggested that the content is made easy to access and understand as possible, written in a common nonacademic language, and made available in smaller sections to avoid overwhelming patients. To increase engagement and adherence, it was considered essential to ensure that all participants could easily download the intervention. Having an actual person, a “human contact,” connected to the technology was also described as a potentially important factor for success. Table 1 summarizes participant comments in the exploration phase of the research.

#### Personas and Journey Map

**Figure 3:**
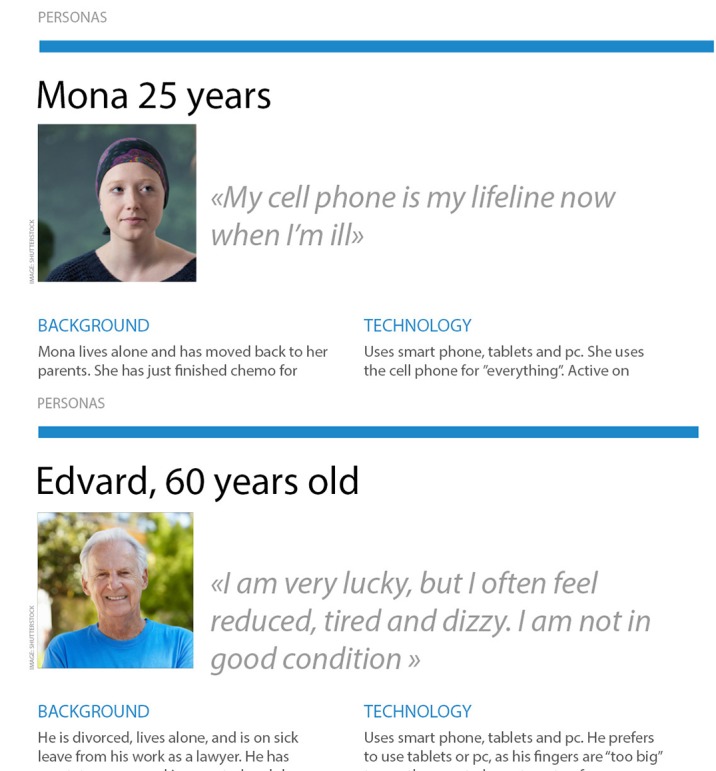
Illustrated examples of personas. PC: personal computer.

Insights from the interviews were used to create Personas. The use of Personas is a method from user-centered and service design utilized to create and visualize fictional representations of the target group [46]. Use of Personas is an effective method for all project team members, particularly for the IT designers and developers, to get an enhanced understanding of the target group that the app is built for. Personas in this study contained information about the cancer survivors’ background and challenges, their use of technology, and their needs and requirements for an electronic stress management intervention, as seen in Figure 2. The Personas were used in the design and development process as a tool to ensure that user voices were taken into account during the design and development phase; See Figure 3 for illustrated examples of study personas.

In addition, a Journey Map (a roadmap visualizing the user interaction with the service) [36] was created based on the interviews and input from the multidisciplinary team with health care providers, eHealth experts, the designer, and IT developers (see Multimedia Appendix 1). The Journey Map was created to visualize a common project understanding, displaying touch points between the user and the intervention (from the user or patient perspective), including all potential contact points with the project team and health care providers during the information stage, inclusion process, app use, and follow-up.

#### Needs and Requirements: Decisions and Deliverables

Based on input from cancer survivors, health care providers, and eHealth experts, the research team decided that the stress management intervention program would be developed as an app made available for tablets and smartphones. This would also facilitate offering a combination of text, sound files, pictures, and a video. Suggestions were solicited to identify an appropriate intervention app name; with many inputs containing the words “stress,” “management,” and “boss” or “professional,” the final name of *StressProffen* was chosen. Given the described cancer survivor difficulties, such as concentration problems and fatigue, it was also decided that the content should be presented in smaller parts, be intuitive, and easy to navigate.

**Table 1:** Exploration phase: user needs and requirements.

Topics of importance to users	Cancer survivors (n=11)	Health care providers (n=8) and eHealth experts (n=9)
Content	Fulfill their needs Accessible Easy to use Intuitive and user friendly Combination of sound files, text, and a video	Small content sections Easy to access and understand Use of common or lay language
Design	Smartphones or tablets preferred	Gender neutral or appeal to male and female cancer survivors alike
Timing and place for intervention delivery	N/A^a^	A while after diagnosis Out-patient clinics Radiation treatment clinics Learning and mastery units Psycho-oncology service units
Engagement and adherence	N/A	Easy to download Offer log-on support Human contact point

^a^N/A: not applicable.

To facilitate individual contact and increase the potential for engagement and adherence, it was also decided that the stress management intervention would include one face-to-face introductory session where participants would also receive help installing the app. It was also decided that participants would receive a follow-up call during the course of the intervention.

### Intervention Content Development

At the base of the *StressProffen* intervention are concepts from well-known cognitive behavioral stress management interventions for cancer patients [8-12], including the Mayo Clinic QoL and Stress Less Interventions [4,13,33], all guided by theoretical models where cognitive, behavioral, social, personal, and environmental factors interact in guiding motivation and behavior.

**Figure 4:**
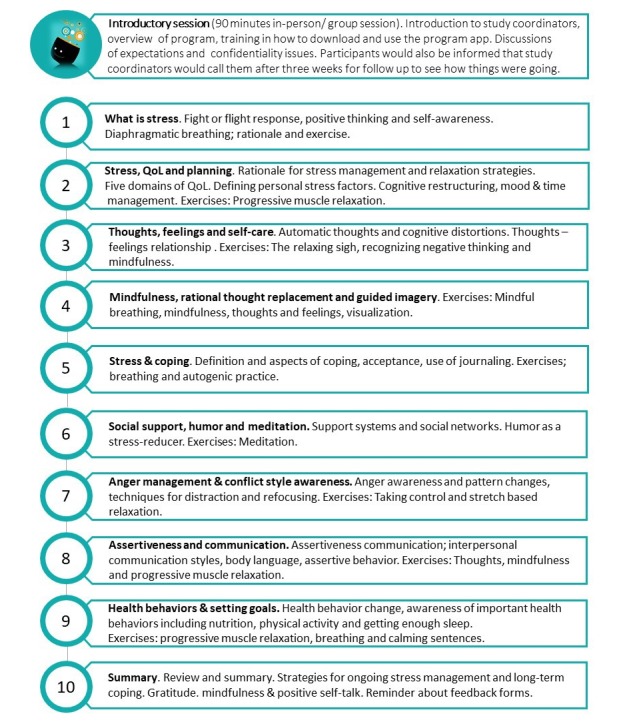
The StressProffen overview of modules and their content. QoL: quality of life.

The actual initial intervention content for this study was first developed by the primary investigator, then adapted and tailored to Norwegian conditions by the entire research team through iterative processes (average 6 iterations per module) to fit a 10-module-based intervention in electronic format through text, sound, video (explaining the fight-or-flight concept), and pictures. Each version was user tested to meet user requirements described above, make the content easily accessible, confirm adaptation to an app format, and ensure that the scientific foundation for the intervention was intact. The iterative content development processes were parallel to app programming, and adjustments were made based on usability testing. The final intervention contained a face-to-face introductory session where *StressProffen* could be downloaded and installed on study participant smartphones or tablets. Figure 4 lists and briefly describes the 10 modules and the topics covered in each module.

### Software Development and Formative Evaluation

**Figure 5:**
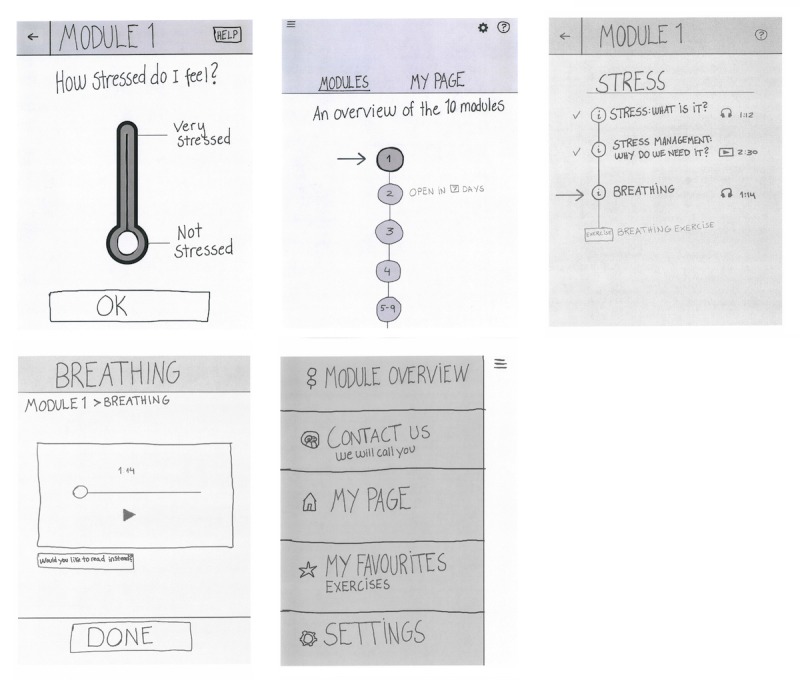
Paper Prototypes from development.

Usability testing of the paper prototype app resulted in adjustments to ensure easier navigation, new icons, and implementation of engaging design to stimulate adherence, adding optional quotes and a more visible option of listening versus reading. In addition, to allow for individual user preferences, it was decided that the app-based program would allow users to mark favorite exercises, which would show up as “My favorites-Exercises.” Individual progress would be available as a part of “My Page,” where participants could find their tracking and progress information, as seen in seen in Figure 5.

Following app programming and complete content implementation, a new set of usability testing and iterations was conducted, as described in the Methods section. Based on user feedback in this phase, the following design recommendation adjustments were made and used to adjust the prototype: information should be stepwise, brief, and short (eg, presented as maximum 3 screens of text); provide information about how much time would be required; all modules should require approximately the same amount of time to complete; type of content (eg, informative, a recommended practice exercise) should be easy to determine by the user; favorite exercises should be easy to locate and access; it should be easy to choose whether one would like to read or listen; the content should be easy to understand and presented in common language with no academic or medical terminology; use of animations and illustrations to create visual aids and substantiate the information in clear and engaging manners; and recorded stress levels should be easy to track in a “My page” option.

**Figure 6:**
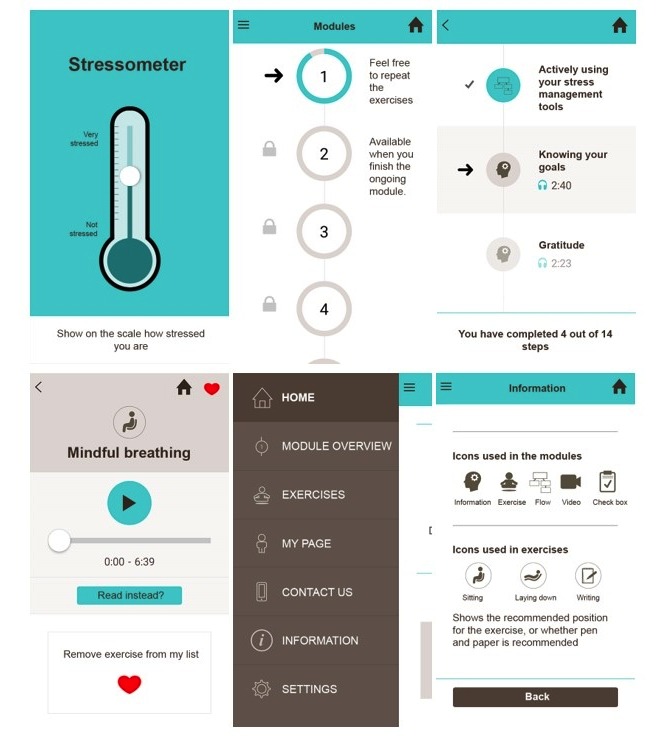
StressProffen app screenshots.

The final version showed the duration of information or exercises (ranging from 1 to 14 minutes), and users could easily see how long each module and section would last, as seen in the screenshot examples in Figure 6.

### Privacy, Security, and Organization Anchoring

#### Security and Privacy Considerations

The *StressProffen* intervention program focuses on stress management for cancer survivors. It was developed at and would be distributed from a major hospital with cancer centers. Therefore, sensitive health-type information had to be carefully considered and protected. When asked about data protection and security, most participants had no concerns. One participant expressed “My life is not that exciting,” and another said “I have nothing to hide.” Protecting patients and patient information is, nevertheless, the responsibility of the hospital and health care professionals, and some of the participants did acknowledge safety concerns and reported being careful about what they posted about their personal information on social media.

To address all security and privacy issues, a risk assessment of the *StressProffen* intervention was evaluated and approved by the hospital Privacy and Security Protection Committee. For example, one security concern was that if the intervention mentioned diagnoses (eg, cancer), this could compromise user privacy. To ensure that patient diagnoses is not revealed if anyone was watching or the phone or tablet lost, one alternative was to ask users for a pin code or password each time they were accessing the app. Even though this measure would protect privacy for the users, such protection could potentially reduce ease of use, which was one of the most important user requirements, and thereby also reduce engagement, adherence, and the potential effect of the intervention. Another option was to completely avoid the word cancer and any cancer-specific information in the app. Based on user input and security recommendations, it was decided to choose the second option and not include diagnosis-specific information.

#### Anchoring the Intervention Within the Organization

To anchor the intervention within the organization, receiving approval from the hospital Privacy and Security Protection Committee was essential. The project was then registered with the hospital innovation unit (ie, *Idépoliklinikken*). The following topics were addressed in the registration: the potential usefulness of the innovation for patients and providers, potential economic impact, a prospective plan for upgrading, and responsibility for running the intervention program after study completion to test intervention effects. After registration, the *StressProffen* app was approved as an official Oslo University Hospital app.

## Discussion

### Principal Findings

This study process identified a variety of stakeholder needs, requirements, and challenges in designing and developing a user-centered evidence- and technology-based stress management intervention program for cancer survivors. Cancer is a major threat to life, health, and well-being [2,4-7], and interviews with cancer survivors in this study underlined this, describing a multitude of stressful daily challenges including fatigue, pain, social isolation, worries, and depression. Targeting these issues through interventions has great potential, but the process of involving stakeholders in intervention design and development is fundamental [37].

Interviews with cancer survivors and feedback from health care providers and eHealth experts in the study gave vital direction for intervention design and development. Cancer survivors preferred stress management through an easy-access user friendly mobile app and identified usefulness, easily understandable language, and brief and to the point sections as other important intervention components. These requirements were also supported by recommendations from health care providers and eHealth experts. Intervention content was rooted in evidence-based cognitive behavioral stress management strategies, synthesized, and adapted to the new *StressProffen* intervention. As an easy-to-access app with evidence-based content in 10 main modules, the intervention was divided into briefer subsections in a variety of readable, auditory, or visual presentations.

### User Requirements

All users stressed the importance of easy access and intuitive programming for them to use a stress management intervention program on an ongoing basis. They also described the importance of using an easily understandable language rather than an academic language or difficult-to-understand medical terminology and stressed the importance of not receiving too much information at once but rather dividing the intervention into smaller, more manageable parts.

A majority of participants preferred using either a tablet or smartphone for stress management because they associated the use of PCs with work. Age appeared to play a role in the preferred choice of device because younger cancer survivor users anticipated preferring smartphones due to easy access, whereas some of the older patient users (>50 years) anticipated preferring to use tablets due to the smaller smartphone screens. Older age has been reported to be a barrier to the uptake of a Web-based intervention for cancer-related distress [47]. However, once enrolled, older individuals have demonstrated better intervention adherence. Adjusting the intervention to fit preferences among different age groups might, therefore, increase uptake and adherence [47].

Several cancer survivors in the study described not having any interest in health apps, with the exception of relaxation apps already used by some of them. Some cancer survivors described searching the internet for health information at times, whereas others stated that they did not want to be scared by all the available information without knowing whether the information “out there” could be trusted. This again pointed to the need for evidence-based information.

### Intervention Content

Rooted in the concept of cognitive behavioral stress management, the final *StressProffen* intervention app contains educational material related to topics such as stress, QoL, planning, thoughts and feelings, coping, social support, anger management, assertiveness and communication, health behaviors, and setting goals. The intervention also contains a variety of exercises, including thought challenges, positive self-talk, diaphragmatic breathing, progressive muscle relaxation, guided imagery, mindfulness, and meditation. Finally, the app also contains a video visualizing and explaining the fight-or-flight concept.

Cancer-specific education material was not included because this would have required a higher privacy and security level (eg, pin code or password log-on), subsequently impacting the user-required aspect of easy access. Such cancer-specific material would also need continuous field-related updates, which would have been labor intensive and potentially complicate implementation. Writing exercises were encouraged, separately from the intervention program, because typing information into the actual app would again require higher privacy and security levels.

### Importance of User Involvement and Evidence

*StressProffen* as a stress management intervention program is rooted in evidence-based methods, a necessity for bringing about change and stress reduction in cancer survivors. Nevertheless, intervention success also depends on whether the intended users consider the app helpful and easy to use [35]. An intervention that is poorly designed, focuses on providing too much or too little information, or complicated to use will not have the intended effect due to low engagement, no matter how evidence-based the stress management content is. The opposite is also true. Although a perfectly designed and pleasurable app may be used a lot, if it is not rooted in evidence, the chance of bringing about positive change is unlikely. Evidence-based strategies, user input, and user-friendly technology need to work in harmony for an app to be widely used and effective.

In this study, using service design and user-centered design methods, involving stakeholders, including cancer survivors, health care providers, and essential organizational units such as the hospital Privacy and Security Protection Committee and the Innovation unit, facilitated the identification of a range of necessary needs and requirements for a potentially effective stress management app for cancer survivors. This process is in line with the Center for eHealth Research and Disease Management comprehensive roadmap approach to improve the uptake and impact of eHealth technologies [37]. The approach recommends multidisciplinary project management in combination with contextual user and environmental inquiry along with iterative design processes with end-user prototype testing to enhance chances of future implementation success.

### Privacy and Security Aspects

An important factor contributing to use is the fact that an app is easy to use and access [35]. The *StressProffen* intervention is developed by and anchored in a large hospital with health-related privacy and security regulations at the forefront. Any information considered sensitive requires a secure user log-in procedure for user access. Therefore, *StressProffen* contains no cancer-specific information and does not allow users to write or store their own notes in the app. As such, the user requirement of easy access was given priority over providing cancer-specific information or advice. It remains to be determined if this decision will be viewed as a weakness or strength by future users. The lack of cancer-specific content might limit the user’s sense of having an individually tailored app, which again might reduce engagement, adherence, and effect.

In contrast, neutralizing content to allow for easy app access through reduced demands for privacy and security can be particularly beneficial for cancer survivors who face challenges such as fatigue and difficulties with memory and concentration. Additionally, in the long run, a more generic stress management program might have a potential benefit for other groups of patients, caregivers, or family members [48].

### Strengths, Limitations, and Future Directions

The study has some limitations that need to be considered. Cancer survivors participating in the user interviews responded to hospital or social media-related invitations and represented a sample of convenience, which can introduce selection bias. However, the cancer survivors were both male and female and represented a wide range of age and cancer diagnoses. As such, the survivors participating in the study were representative of future potential app users. The usability testing methods in this study may also present with limitations. First, usability testing involved all functionalities but not all content sentences. Second, the usability testing included a limited number of participants, which is, however, not uncommon for iterative design processes [49]. Based on the above, it is possible that the usability testing of *StressProffen* so far captured only some of the potential barriers to continuous use over time. This will need to be further addressed in ongoing user research. Accordingly, to refine the *StressProffen* intervention, a feasibility pilot is planned where a larger number of participants will test the entire 10-module intervention in their own environment, complete outcome measures to gauge preliminary effects, and participate in qualitative interviews to elaborate on user experiences. User log data will also be extracted to observe actual use.

This design and development study also has a number of strengths. First, the study employed key stakeholder involvement from the very beginning, including cancer survivors, health care providers working with survivors in various hospital units, eHealth experts including a designer and IT developers, the hospital Privacy and Security Protection Committee, and the Innovation unit. Having user involvement from the start and combining input obtained from the patient user with that obtained from health care providers along with evidence-based concepts and content likely increases the potential for the intervention to be effective. Also, early stakeholder involvement, including ensuring privacy and security requirements and anchoring the intervention in the organization, may increase the potential for poststudy implementation.

### Conclusions

Intervention programs, even evidence-based, have at best limited impact if not actively used over time. The ultimate goal of the *StressProffen* intervention is to have an end product that is both user friendly and useful, engaging and motivating, and fits into the bigger context of the everyday life and challenges of people living with cancer. Even though the user-centered design process can be labor intensive, time consuming, and as such also costly, it is likely a waste of resources not to invest enough time and effort in the essential design and development phase. This study illustrates how user-centered design and service design approaches can identify and incorporate vital user and stakeholder aspects in the early design phase and then in combination with evidence-based concepts facilitate the development of a stress management intervention truly designed for the end users, in this case, people living with cancer.

## Appendix

Multimedia Appendix 1 Journey map.

## Abbreviations

IT: information technology
PC: personal computer
QoL: quality of life
