# Supplementary figures and images for: A Stress Management App Intervention for Cancer Survivors: Design, Development, and Usability Testing
Source: JMIR Form Res. 2018 Sep 6;2(2):e19. doi: 10.2196/formative.9954 (PMC6334690; doi:10.2196/formative.9954)

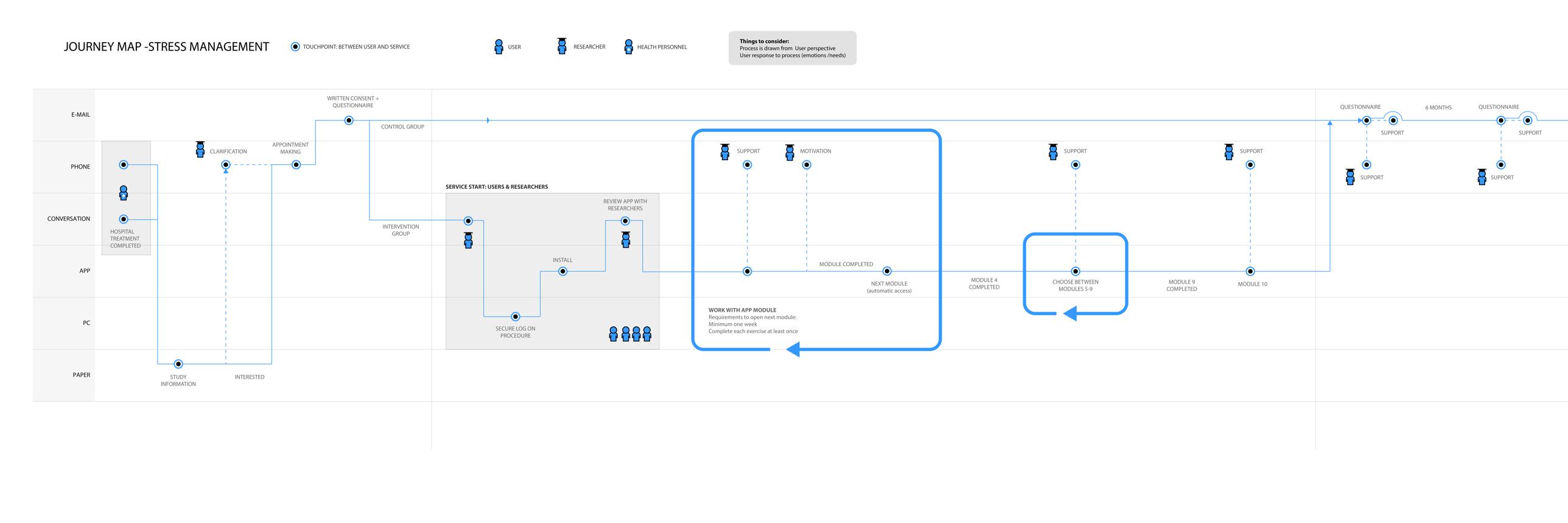

Supplement: Multimedia Appendix 1 [file formative_v2i2e19_app1.PNG]
